# Crystal structure of endo-1,4-β-glucanase from *Eisenia fetida*

**DOI:** 10.1107/S0909049513021110

**Published:** 2013-10-01

**Authors:** Takao Arimori, Akihiro Ito, Masami Nakazawa, Mitsuhiro Ueda, Taro Tamada

**Affiliations:** a Quantum Beam Science Directorate, Japan Atomic Energy Agency, 2-4 Shirakata-Shirane, Tokai, Ibaraki 319-1195, Japan; b Graduate School of Life and Environmental Sciences, Osaka Prefecture University, 1-1 Gakuen-cho, Sakai, Osaka 599-8531, Japan

**Keywords:** endoglucanase, glycoside hydrolase family 9, cold-adapted enzyme, *Eisenia fetida*

## Abstract

The saccharification process is essential for bioethanol production from woody biomass including celluloses. Cold-adapted cellulase, which has sufficient activity at low temperature (<293 K), is capable of reducing heating costs during the saccharification process and is suitable for simultaneous saccharification and fermentation. Endo-1,4-β-glucanase from the earthworm *Eisenia fetida* (EF-EG2) belonging to glycoside hydrolase family 9 has been shown to have the highest activity at 313 K, and also retained a comparatively high activity at 283 K. The recombinant EF-EG2 was purified expressed in *Pichia pastoris,* and then grew needle-shaped crystals with dimensions of 0.02 × 0.02 × 1 mm. The crystals belonged to the space group *P*3_2_21 with unit-cell parameters of *a* = *b* = 136 Å, *c* = 55.0 Å. The final model of EF-EG2, including 435 residues, two ions, seven crystallization reagents and 696 waters, was refined to a crystallographic *R*-factor of 14.7% (free *R*-factor of 16.8%) to 1.5 Å resolution. The overall structure of EF-EG2 has an (α/α)_6_ barrel fold which contains a putative active-site cleft and a negatively charged surface. This structural information helps us understand the catalytic and cold adaptation mechanisms of EF-EG2.

## Abstract 2

The crystal structure of endo-1,4-β-glucanase from the earthworm *Eisenia fetida*, which retained sufficient cellulase activity at low temperature, was determined at 1.5 Å resolution.

## 1. Introduction

Cellulose is the most abundant organic molecule on earth and is an excellent target for bioethanol production by biomass conversion. Bioethanol production from plant-derived lignocellulosic wastes would effectively reduce food costs relative to that from corn or cane juice (Dashtban *et al.*, 2009 ▶). Most process concepts for bioethanol from lignocellulosic material start with a thermo-chemical hydrolysis of the hemicelluloses part (pretreatment), followed by an enzymatic hydrolysis of the cellulose part (sacccharification) and yeast-based fermentation of the resulting sugar. Fungi, bacteria and invertebrates produce enzymes named cellulases that are capable of degrading the linear biopolymers of anhydro­glucopyranose connected by β-1,4-glycosidic bonds into sugars. In the pretreatment process, the lignocellulosic materials are heated over 373 K in the alkaline or acidic solution to expose the cellulose. The use of thermophilic fungal species, such as *Sporotrichum thermophile* and *Thielavia terrestris*, can avoid a costly cooling step after the pretreatment process (Kovacs *et al.*, 2009 ▶; Ingram *et al.*, 2011 ▶). Instead, the reaction temperature must be kept above 333 K by a heating step to maintain its enzymatic activity at the saccharification process. Furthermore, such high temperature is unsuitable for the adaptation of the simultaneous saccharification and fermentation (SSF) process. In the SSF process, the enzymatic hydrolysis is performed together with the fermentation. The principal benefits of the SSF process are the reduced end-product inhibition of the enzymatic hydrolysis, and the reduced investment costs. In addition, SSF is capable of the production of a high concentration of ethanol (>20%). Thus, SSF is today important in the dry-milling process in the corn-based ethanol industry in the USA (Bothast & Schlicher, 2005 ▶), and is also an interesting process option for bioethanol production from lignocelluloses (Olofsson *et al.*, 2008 ▶). Unfortunately, the enzymatic activity of most thermophilic organisms is almost lost at the optimal temperature (298–303 K) in the SSF process.

Recently, we cloned the gene for endo-1,4-β-glucanase from the earthworm *Eisenia fetida* (EF-EG2), which consisted of 1368 bp encoding 456 amino acid residues (Ueda *et al.*, 2013 ▶). The amino acid sequence of the gene shares sequence homology (>50%) with endo-1,4-β-glucanases belonging to glycoside hydrolase (GH) family 9. Recombinant EF-EG2 hydrolyzes soluble cellulose (carboxymethyl cellulose), but not insoluble (powdered cellulose) or crystalline (Avicel) cellulose substrates. Thin-layer chromatography analysis of the reaction products from 1,4-β-linked oligosaccharides of various lengths revealed a cleavage mechanism consistent with endoglucanases (not exoglucanases). The enzyme exhibited significant activity at 283 K (38% of the activity at optimal 313 K) and was stable at pH 5.0–9.0, with an optimum pH of 5.5. However, the catalytic mechanism and the cold adaptation mechanism of EF-EG2 are still unknown. Here we report the first crystal structures of EF-EG2. Structural information of EF-EG2 provided useful information for understanding its catalytic mechanism by comparing it with other GH family 9 enzymes. In addition, we discuss the cold adaptation mechanism of EF-EG2 with regard to its structural features.

## 2. Methods

### 2.1. Expression and purification

Forward (5′-ggctgaagctgaattccaatataattatgacgaagttc-3′, *Eco*RI site underlined) and reverse (5′-gagtttttgttctagaaacttgccgtc­tctcagctgaat-3′, *Xba*I site underlined) primers were synthesized on region corresponding to amino acid residues 22–29 and 450–456 of EF-EG2, respectively. The PCR reaction mixture (50 µl Prime STAR buffer) contained *E. fetida* cDNA, 0.25 µ*M* of each primer, 200 µ*M* of each dNTP and 1.25 U of Takara Prime STAR DNA polymerase (Takara Bio). The amplified fragment was inserted into the *Eco*RI/*Xba*I sites of the pPICZαA vector (Invitrogen). The expression plasmid was linearized by *Sac*I and transformed into the competent *Pichia pastoris* GS115 cells by electroporation. Cells were spread on YPDS medium (1% yeast extract, 2% peptone, 2% dextrose, 1 *M* sorbitol, 1.5% agar) containing 100 µg ml^−1^ of Zeocin and incubated at 301 K for 2–4 d. Colonies were picked up and spread onto each YPDS plate containing 100, 500, 1000, 2000 mg ml^−1^ of Zeocin. Colonies capable of growth in the presence of a high concentration of Zeocin were then selected. The selected colonies were cultured in a 500 ml Erlenmeyer flask containing 25 ml BMGY medium (1% yeast extract, 2% peptone, 100 m*M* potassium phosphate pH 6.0, 1.34% YNB, 4 × 10^−5^% biotin, 1% glycerol) at 301 K at 250 r.p.m. for 48 h. The culture media was centrifuged at 3000*g* for 5 min and resulting cell pellets were resuspended in BMMY medium (1% yeast extract, 2% peptone, 100 m*M* potassium phosphate pH 6.0, 1.34% YNB, 4 × 10^−5^% biotin, 0.5% methanol). The cell suspension was then added to a jar-fermenter containing 2.9 l of BMMY medium and grown at 290 K for 7 d at 300 r.p.m. for aeration at 3 l min^−1^ during which 0.5% methanol was added daily.

After cultivation, the culture media was centrifuged at 8200*g* for 10 min at 277 K, before the supernatant was recovered. EF-EG2 was precipitated from the supernatant with ammonium sulfate (80% saturation) followed by centrifugation at 20000*g* for 30 min. The precipitate was dissolved in 20 m*M* Tris–HCl buffer (pH 7.5) containing a Protease Inhibitor Cocktail (Nacalai Tesque). EF-EG2 was purified using a Superdex 75 gel filtration column (GE Healthcare) equilibrated with 20 m*M* HEPES buffer (pH 7.5) containing 200 m*M* sodium chloride. The eluted fraction containing EF-EG2 was diluted with the same amount of 20 m*M* Tris-HCl buffer (pH 7.5), and applied to a RESOURCE Q anion-exchange column (1 ml; GE Healthcare). After the column had been washed with 10 ml of 20 m*M* Tris–HCl buffer (pH 7.5) containing 50 m*M* sodium chloride, the EF-EG2 was eluted with a linear gradient from 20 m*M* Tris-HCl buffer (pH 7.5) containing 50 m*M* sodium chloride to the same buffer containing 400 m*M* sodium chloride with a flow rate of 1.0 ml min^−1^ for 20 min. Purified EF-EG2 was concentrated to 5.5 mg ml^−1^ for crystallization.

### 2.2. Crystallization

Initial screening for EF-EG2 crystallization was performed by the sitting-drop vapour-diffusion method at 293 K using 96-well Intelliplates (Hampton Research) and a Hydra II Plus One (Matrix Technology). Each sitting drop was prepared by mixing 0.3 µl of the protein solution and of reservoir solution; the resulting drop was equilibrated against the reservoir solution. The initial search for crystallization conditions was performed using the following screening kits: Crystal Screen I and II (Hampton Research), Wizard Screen I and II (Emerald Biostructures) and Precipitant Synergy (100, 67 and 33% of its primary concentration; Emerald Biostructures). After 10 d, small crystals were obtained in some conditions. Crystallization conditions were further optimized by changing the precipitant based on the conditions from Wizard Screen II No. 31 (200 m*M* sodium chloride, 1 *M* sodium citrate, 100 m*M* Tris-HCl pH 7.0). The optimization of crystallization conditions was performed by using the hanging-drop vapour-diffusion method at 293 K. Needle-shaped crystals with dimensions of 0.02 × 0.02 × 1 mm were grown from a drop consisting of 2.0 µl each of the protein solution and the reservoir solution containing 200 m*M* sodium chloride, 600 m*M* sodium citrate and 67 m*M* Tris-HCl pH 7.0.

### 2.3. Data collection and refinement

For X-ray diffraction measurements under cryogenic conditions, EF-EG2 crystals were rinsed with the well solution containing 20% (*v*/*v*) glycerol as a cryo-protectant and then flash-cooled in a cold nitrogen gas stream. X-ray diffraction data from the crystal were collected at 100 K using an ADSC Quantum 315r CCD detector (Area Detector Systems Co., CA, USA) and synchrotron radiation (0.98 Å wavelength) at beamline BL17A at Photon Factory, KEK (Tsukuba, Japan). The oscillation angle was 1.0° and the exposure time was 2.0 s per frame. In total, 180 diffraction images were recorded at a camera distance of 155.4 mm and were processed using *HKL2000* (Otwinowski & Minor, 1997 ▶) to 1.5 Å resolution. The crystal belonged to the space group *P*3_2_21 with unit-cell dimensions *a* = *b* = 136, *c* = 55.0 Å. The Matthews coefficient was 2.9 Å^3^ Da^−1^ assuming that the presence of one molecule in the asymmetric unit corresponded to the solvent content of 57%.

Initial phase information was obtained by molecular replacement analysis with *PHASER* (McCoy *et al.*, 2007 ▶) using the coordinates of endoglucanase from *Nasutitermes takasagoensis* [NtEgl; Protein Data Bank (PDB) ID: 1ksd] (Khademi *et al.*, 2002 ▶), which has 56% sequence identity with EF-EG2. The atomic model was built and modified with *COOT* (Emsley & Cowtan, 2004 ▶). The final model structure of EF-EG2 was refined to a crystallographic *R*-factor of 14.7% (free *R*-factor of 16.8%) to 1.5 Å resolution using restrained refinement in *REFMAC* (Murshudov *et al.*, 1997 ▶). The Ramachandran conformational parameters from the last cycle of refinement generated by *RAMPAGE* (Lovell *et al.*, 2003 ▶) showed that 97.5% of the residues in the EF-EG2 structure had the most favoured conformation with one residue (Gly168) in the outlier region, even though its corresponding electron densities were clear. Statistics of data collection and refinement are summarized in Table 1 ▶.

## 3. Results and discussion

### 3.1. Crystal structure of EF-EG2

**Figure 1:**
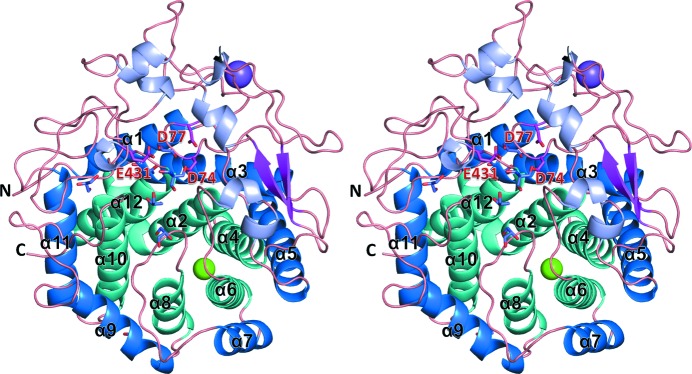
Overall structure of EF-EG2. Inner and outer helices of the (α/α)_6_ barrel, other short helices and β-strands are colored in cyan, blue, light purple and magenta, respectively. Crystallization reagents and putative active residues are shown as purple and magenta stick models, respectively. Two ions, calcium and sodium, are shown as green and purple sphere models, respectively. All figures were prepared using the *PyMOL* Molecular Graphics System (DeLano Scientific, San Carlos, CA, USA).

The crystal structure of EF-EG2 was determined at 1.5 Å resolution as the second example of eukaryotic GH family 9 enzyme following that of NtEgl. The final refined EF-EG2 structure includes 435 residues (Phe21–Gly455), which have 23 alternative side-chain conformations, two ions (Ca^2+^ and Na^+^), seven crystallization reagents (one citrate, one Tris and five glycerol molecules) and 694 waters. The overall structure of EF-EG2 consists of 12 α-helices which form the (α/α)_6_ barrel fold, an additional six short helices and two β-strands (Fig. 1 ▶). The inner six helices, α2 (Gly82–Ala106), α4 (Ser169–Arg187), α6 (Tyr231–Thr246), α8 (Lys273–Ala285), α10 (Pro322–Tyr339) and α12 (Asn434–Leu452), form a central barrel through hydrophobic interactions. The outer six helices, α1 (Tyr24–Glu39), α3 (Gln108–Ala125), α5 (Ala191–Asn210), α7 (Gln249–Phe258), α9 (Thr288–Trp301) and α11 (Ala343–Leu358), surround the inner barrel. The (α/α)_6_ barrel motif is a typical folding pattern of GH family 9 enzymes (Juy *et al.*, 1992 ▶; Sakon *et al.*, 1997 ▶). A structure similarity search using *DALI* (http://www.ebi.ac.uk/dali) (Holm & Rosenström, 2010 ▶) indicated that all 11 highest-scoring (*Z* > 20) structures belong to GH family 9, and the overall structure of EF-EG2 is similar to other GH family 9 structures with root-mean-square deviation values of 1.0–3.0 Å over 350 corresponding Cα atoms.

**Figure 2:**
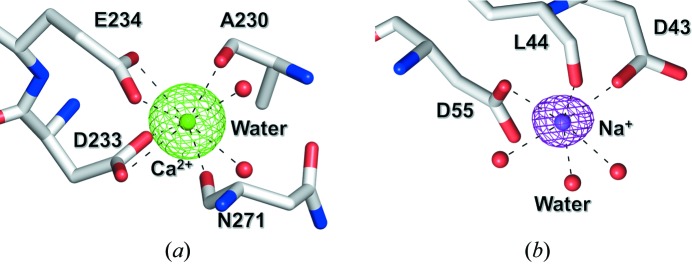
Binding sites of calcium (*a*) and sodium (*b*) ions in EF-EG2 structure. The green and purple contours show the *F*_o_ − *F*_c_ (7.0σ) map calculated without calcium and sodium ions, respectively. Each binding site is composed of eight and six oxygen atoms chelating to a calcium and sodium ion with distances of 2.3–2.5 and 2.3–2.7 Å (represented by black dashed lines), respectively.

A calcium ion located at the N-terminal site of the inner barrel is coordinated to two waters (2.3 and 2.4 Å), two main-chain carbonyl groups (O-Ala230, O-Asn271: 2.5 and 2.4 Å) and two side-chain carboxyl groups (Oδ1, Oδ2-Asp233: 2.5 and 2.5 Å, O∊1, O∊2-Glu234: 2.5 and 2.5 Å) in a square antiprism geometry (Fig. 2*a* ▶). The location of the calcium ion is conserved in GH family 9 enzyme structures; however, its role in the catalytic activities of these enzymes is still unclear. A sodium ion, probably induced by the reservoir solution containing 200 m*M* sodium chloride and 600 m*M* sodium citrate, exists in the loop region between α1 and α2 with a triangular antiprism geometry formed by three waters (2.3, 2.4 and 2.7 Å), a main-chain carbonyl group (O-Leu44: 2.4 Å) and two side-chain carboxyl groups (Oδ1-Asp43, Oδ1-Asp55: 2.4 and 2.4 Å) (Fig. 2*b* ▶). These aspartic acids are conserved in endoglucanase Cel9G from *Clostridium cellulolyticum* (Mandelman *et al.*, 2003 ▶) and also participate in the recognition of magnesium ions included in the crystallization medium. In addition, several clear electron densities were interpreted as a citrate (precipitant), a Tris (buffer) and five glycerol molecules (cryo-protectant).

### 3.2. Active site

**Figure 3:**
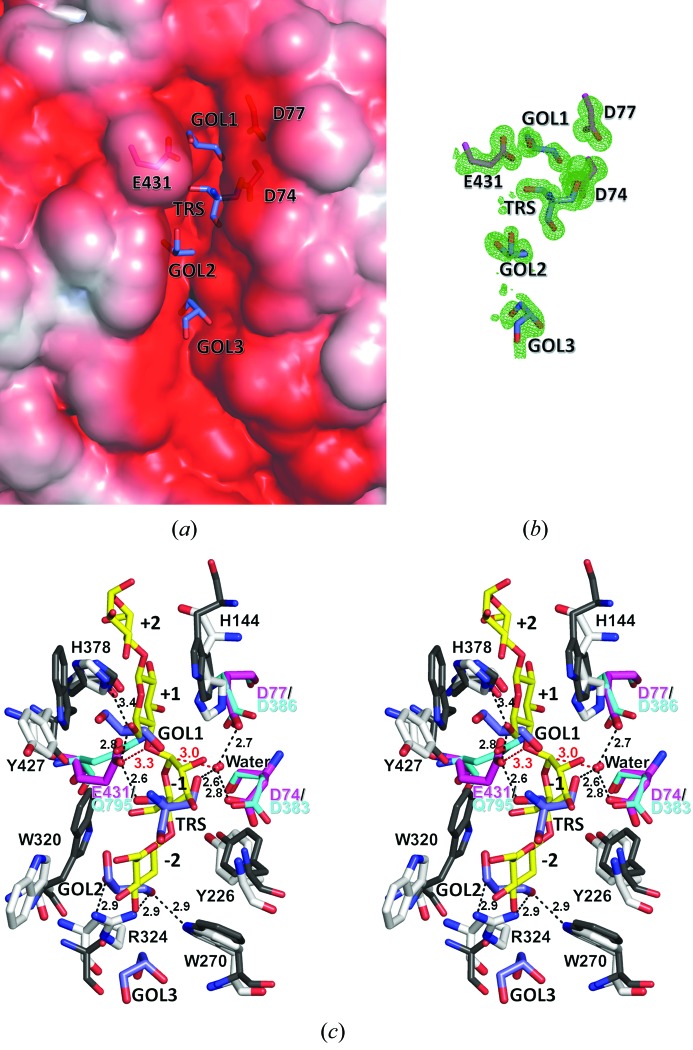
Putative active site of EF-EG2. (*a*) Solvent accessible surface showing the binding cleft. The surface is colored according to the local electrostatic potential as calculated by *APBS* (Baker *et al.*, 2001 ▶) from −3*kT* (red) to +3*kT* (blue). (*a*) is drawn from the identical direction of Fig. 1 ▶ and is a close-up view of the left-hand side of Fig. 4 ▶(*a*). (*b*) Electron densities of putative active residues and crystal reagents at the binding cleft. The dark green contour shows the *F*_o_ − *F*_c_ (3.5σ) map calculated without TRS, GOL1, 2, 3 molecules and the side-chain atoms Asp74, Asp77 and Glu431. (*c*) Superposition of residues Asp74, Asp77 and Glu431 from EF-EG2 (magenta) on Asp383, Asp386 and Gln795 from CbhA (cyan). Residues within 4 Å from TRS, GOL1, GOL2 (in EF-EG2 structure, purple) and CTT (in CbhA structure, yellow) are shown as white and gray stick models, respectively. Hydrogen-bonding interactions (within 3.4 Å) in the EF-EG2 structure are represented by black dashed lines. Bond lengths that may be useful for elucidating the catalytic mechanism of EF-EG2 are indicated by red dashed lines.

Out of the above crystallization reagents, a Tris (TRS) and three glycerol molecules (GOL1, GOL2 and GOL3) were confirmed with sufficient electron densities at the open acidic cleft located at the N-terminal site of the inner barrel [Figs. 3(*a*) and 3(*b*) ▶]. The volume of this cleft was calculated to be 320 Å^3^ (about 25 Å long, 4–6 Å wide and 6–8 Å deep) using *POCASA* (http://altair.sci.hokudai.ac.jp/g6/index-e.html) (Yu *et al.*, 2010 ▶). This acidic cleft has been widely known as an active-site cleft in GH family 9 enzymes. Fourteen structures (six enzymes) out of 28 structures (11 enzymes) deposited in the PDB have been determined as complex structures with glucopyranose molecules (Sakon *et al.*, 1997 ▶; Parsiegla *et al.*, 2002 ▶; Mandelman *et al.*, 2003 ▶; Schubot *et al.*, 2004 ▶; Pereira *et al.*, 2009 ▶; Eckert *et al.*, 2009 ▶; Moréra *et al.*, 2011 ▶), and in all 14 structures glucopyranose molecules locate in this cleft. Recognition residues of Tris and glycerols are shown in Fig. 3(*c*) ▶. Several aromatic residues, His144, Tyr226, Trp270, Trp320, His378 and Tyr427, along the cleft were situated within 4 Å of the Tris and glycerol molecules. In addition, some hydrogen-bonding interactions contribute to recognition of these molecules. Three hydrogen bonds are constructed, between hydroxyl oxygen atoms of GOL2 and the side-chain nitrogen atom of Trp270 and Arg324, with an N—O distance of 2.9 Å. The hydroxyl oxygen atom of GOL1 is 3.4 and 2.8 Å distant from the side-chain nitrogen and oxygen atoms of His378 and Glu431, respectively. TRS is recognized by the side-chain carboxyl oxygen atom of Glu431 *via* direct hydrogen bond (O—O distance: 2.6 Å) and by those of the Asp74 and Asp77 *via* water-mediated hydrogen bonds. These glutamic and aspartic acid residues, Glu431, Asp74 and Asp77, are conserved throughout GH family 9 enzymes, and are thought to be crucial residues for its catalytic activity. These three acidic residues are located in the upper limb of the cleft (Fig. 3*a* ▶).

To understand the catalytic mechanism of EF-EG2, the crystal structure of an inactive mutant (E795Q) of cellobiohydrolase CbhA from *Clostridium thermocellum* in complex with cellotetraose (CTT) (Schubot *et al.*, 2004 ▶) was superimposed on the EF-EG2 structure at three Cα atoms of putative catalytic residues (Fig. 3*c* ▶). In superposed structures, the location of TRS, GOL1 and GOL2 loosely corresponded to the glucose units in subsites −1, +1 and −2, respectively. This consistency is not surprising because we already confirmed experimentally that glycerols (cryo-protectants) and Tris or HEPES molecules (buffer components) occupy a similar position as the substrate, *N*-acetyl-d-glucosamine residues, in the crystal structures of chitinase C from *Ralstonia sp*. A-471 (Arimori *et al.*, 2013 ▶). This superposed model structure gave us useful information about the catalytic mechanism of EF-EG2. The side-chain carboxyl group of Glu431 may exist within the possible distance [red dashed line in Fig. 3(*c*) ▶] to donate a proton to the scissile bond as a general acid in the inverting mechanism. On the other hand, a water molecule, which is located at the same position as the catalytic water in the superposed structure of the CbhA–CTT complex, may be situated close to the C1 atom to be suitable for nucleophilic attack [red dashed line in Fig. 3(*c*) ▶]. One of two aspartic acid residues situated within hydrogen-bonding distance may activate catalytic water as a general base. Furthermore, the aromatic residues around Tris and glycerol molecules are conserved as aromatic amino acids in CbhA. Some aromatic residues in the EF-EG2 structure may also play a role as possible partners for stacking interactions with the substrate. These hypotheses on the catalytic and substrate recognition mechanism of EF-EG2 will have to be tested through a combination of structural and mutation studies.

### 3.3. Cold adaptation

Structural features of cold-adapted enzymes have been summarized in a review by Siddiqui & Cavicchioli (2006 ▶). The article concludes that high structural flexibility, particularly around the active site, is translated into low-activation enthalpy, low-substrate affinity and high specific activity at low temperatures. The high flexibility is also accompanied by a trade-off in stability, resulting in heat lability. There was no remarkable structural flexibility at the active site because ten residues, Asp74, Asp77, His144, Tyr226, Trp270, Trp320, His378, Tyr427 and Glu431 [shown in Fig. 3(*c*) ▶] which appear to concern enzymatic activity have almost the same average *B*-factor (7.3 Å^2^) as that of the overall structure (8.3 Å^2^). In their review, overall structural features correlated with high flexibility. These structural features are: (i) high surface charge, particularly negative charge; (ii) few electrostatic interactions, particularly few arginine-mediated interactions; (iii) few hydrophobic interactions, particularly few aromatic–aromatic interactions; (iv) secondary structure elements, such as weak intrahelical charge–dipole interactions, and the existence of a proline residue as a helix breaker; (v) surface loops with few prolines and many glycines; (vi) other factors, such as bridges *via* metal ion and disulfide bonds.

**Figure 4:**
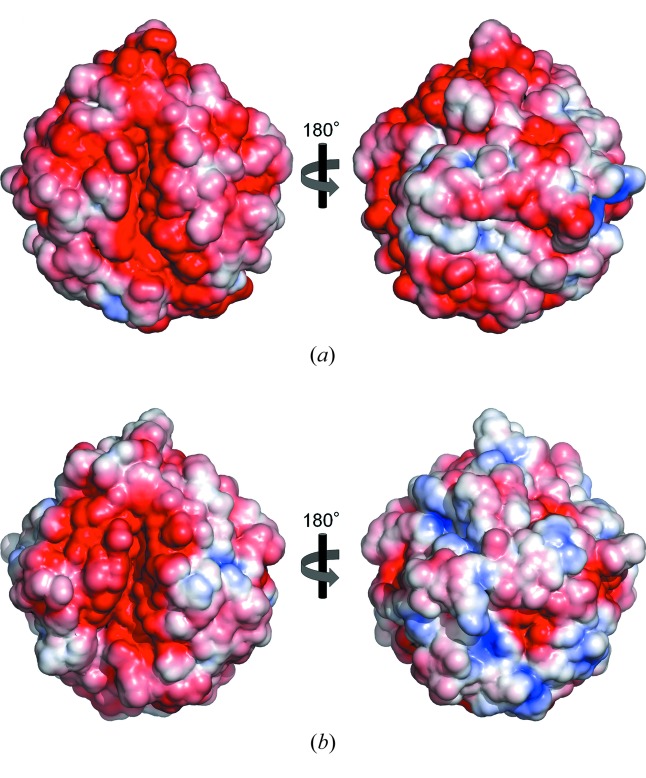
Electrostatic potentials of (*a*) EF-EG2 and (*b*) NtEgl. Solvent accessible surfaces are contoured from −3*kT* (red) to +3 *kT* (blue).

Because of these structural features, we compared the structure of EF-EG2 with that of NtEgl from *N. takasagoensis*, which live in subtropical zones. NtEgl has an optimal activity at 340 K and loses 47% activity at 303 K (Kesavulu *et al.*, 2012 ▶). There was no significant difference between both structures with regard to features (ii)–(vi) listed above. However, there was a slight difference in molecular surface charge (Fig. 4 ▶). The substrate binding face is negatively charged in both structures (left-hand figures); in contrast, the distribution of negative charge at the opposite face (right-hand figures) of EF-EG2 is larger than that of NtEgl. The negatively charged amino acids (Asp and Glu) of EF-EG2 occupy over two-thirds of accessible surface area (ASA) from the total charged amino acids (ASA_Asp,Glu_: 2900 Å^2^; ASA_Arg,Lys_: 1300 Å^2^) whereas those of NtEgl occupy about half (ASA_Asp,Glu_: 2400 Å^2^; ASA_Arg,Lys_: 2300 Å^2^). In addition, those of the *Clostridium thermocellum* cellulase CelT (CtCelT) which has optimal activity at 343 K and retains 72% activity at 303 K (Kesavulu *et al.*, 2012 ▶) occupy about half (ASA_Asp,Glu_: 2900 Å^2^; ASA_Arg,Lys_: 3200 Å^2^). CtCelT has a slightly higher activity at 303 K than NtEgl, but almost loses activity at 293 K. Thus, it seems that the highly negatively charged surface of EF-EG2 contributes to its cold adaptation under 293 K. The increase in surface negative charge has also been described for cold-adapted cellulase belonging to GH family 5 from the Antarctic bacterium *Pseudoalteromonas haloplanktis* which retains sufficient activity at 297 K (Garsoux *et al.*, 2004 ▶).

At low temperature, the energy cost of breaking the hydrogen-bonding network is very high because of the high viscosity and high surface tension of water (Kumar & Nussinov, 2004 ▶). Therefore, the energetic cost may be cancelled out by the surface charge of acidic amino acids interacting with water molecules, accordingly maintaining overall structural flexibility. In addition, the localization of acidic residues in the surface may produce charge–charge repulsions causing destabilization of overall structure. This low structural stability caused by a lop-sided negatively charged surface in cold-adapted enzymes may mainly contribute to maintain its activity at low temperature.

## Supplementary Material

PDB reference: 3wc3

## Figures and Tables

**Table 1:** Data collection and refinement statistics of EF-EG2 Values in parentheses are for the highest-resolution shell.

Data collection	
Space group	*P*3_2_21
Unit cell (Å)	*a* = *b* = 136, *c* = 55.0
Resolution (Å)	32.0–1.50 (1.55–1.50)
No. of measured reflections	760392
No. of unique reflections	92837 (9061)
Multiplicity	8.2 (6.3)
〈*I*/σ(*I*)〉	30.4 (9.0)
*R*_merge_ †	0.099 (0.356)
Completeness (%)	98.8 (97.1)

Refinement	
Resolution (Å)	28.2–1.50 (1.54–1.50)
No. of used reflections	92643
*R*_work_ ‡	0.147 (0.234)
*R*_free_ §	0.168 (0.260)
No. of protein residues	435
No. of ions/crystallization reagents	2/7
No. of water molecules	694
Average *B*-factor (Å^2^)	11.2
Protein	8.3
Ion/crystallization reagent	11.2/24.2
Water	25.0
Root mean square deviations	
Bond length (Å)	0.010
Bond angles (°)	1.24

† *R*_merge_ = Σ_*hkl*_Σ_*i*_|*I*_*i*_(*hkl*) − 〈*I*(*hkl*)〉|Σ_*hkl*_Σ_*i*_ *I*_*i*_(*hkl*).

‡ *R*_work_ = Σ||*F*_obs_| − |*F*_calc_||/Σ|*F*_obs_|.

§ *R*_free_ is calculated with 5% of data omitted from the refinement.
